# Adaptive Variation in Beach Mice Produced by Two Interacting Pigmentation Genes

**DOI:** 10.1371/journal.pbio.0050219

**Published:** 2007-08-14

**Authors:** Cynthia C Steiner, Jesse N Weber, Hopi E Hoekstra

**Affiliations:** 1 Division of Biological Sciences, University of California San Diego, La Jolla, California, United States of America; 2 Department of Organismic and Evolutionary Biology, Harvard University, Cambridge, Massachusetts, United States of America; 3 Museum of Comparative Zoology, Harvard University, Cambridge, Massachusetts, United States of America; Duke University, United States of America

## Abstract

Little is known about the genetic basis of ecologically important morphological variation such as the diverse color patterns of mammals. Here we identify genetic changes contributing to an adaptive difference in color pattern between two subspecies of oldfield mice *(Peromyscus polionotus)*. One mainland subspecies has a cryptic dark brown dorsal coat, while a younger beach-dwelling subspecies has a lighter coat produced by natural selection for camouflage on pale coastal sand dunes. Using genome-wide linkage mapping, we identified three chromosomal regions (two of major and one of minor effect) associated with differences in pigmentation traits. Two candidate genes, the *melanocortin-1 receptor (Mc1r)* and its antagonist, the *Agouti signaling protein (Agouti),* map to independent regions that together are responsible for most of the difference in pigmentation between subspecies. A derived mutation in the coding region of *Mc1r,* rather than change in its expression level, contributes to light pigmentation. Conversely, beach mice have a derived increase in *Agouti* mRNA expression but no changes in protein sequence. These two genes also interact epistatically: the phenotypic effects of *Mc1r* are visible only in genetic backgrounds containing the derived *Agouti* allele. These results demonstrate that cryptic coloration can be based largely on a few interacting genes of major effect.

## Author Summary

The tremendous amount of variation in color patterns among organisms helps individuals survive and reproduce in the wild, yet we know surprisingly little about the genes that produce these adaptive patterns. Here we used a genomic analysis to uncover the molecular basis of a pale color pattern that camouflages beach mice inhabiting the sandy dunes of Florida's coast from predators. We identified two pigmentation genes, the melanocortin-1 receptor *(Mc1r)* and its ligand, the agouti signaling protein *(Agouti),* which together produce a light color pattern. We show that this light pigmentation results partly from a single amino acid mutation in *Mc1r,* which reduces the activity of the receptor but does not affect the gene's expression level, and partly from the derived *Agouti* allele, which shows no change in protein sequence but does exhibit an increase in mRNA expression. We also show that these two genes do not act additively to produce pale color; rather, the derived *Agouti* allele must be present to see any effect of *Mc1r* on pigmentation. Thus, the light color pattern of beach mice largely results from the physical interaction between a structural change in a receptor (reducing *Mc1r* activity) and a regulatory change in the receptor's antagonist (increasing *Agouti* expression).

## Abstract 3

Species of oldfield mice have coat colors adapted for their environment. By using genome-wide linkage mapping, the authors show that three chromosomal regions are associated with differences in pigmentation traits.

## Introduction

Animal pigmentation has attracted substantial evolutionary interest because changes in color, be they driven by natural or sexual selection, can have profound effects on fitness. Dissecting the genetic basis of morphological variation, such as adaptive pigmentation, allows us to answer several long-standing evolutionary questions: How many genes contribute to adaptive phenotypes? What are the relative sizes of their effects? Are adaptive alleles generally dominant, semidominant, or recessive? What types of genes are involved in adaptive change? Do adaptive mutations generally occur in coding or regulatory regions? What is the role of epistasis in evolutionary change?

To understand the genetic processes involved in generating adaptive color patterns, we revisited a series of classic natural history studies [1–3] that described geographic variation in coat-color pattern of the oldfield mouse *(P. polionotus)*. The extreme coat-color variation within this species is driven by selection for camouflage [4], yielding a strong geographical correlation between coat color and reflectance of the substrate [5,6].

**Figure 1:**
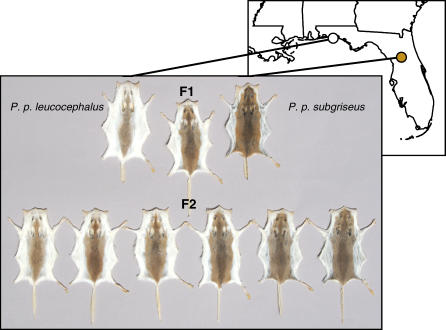
Pigmentation Patterns of a Beach Mouse *(P. polionotus leucocephalus),* a Mainland Mouse *(P. p. subgriseus),* the Resulting Hybrid (F_1_), and the Second-Generation Reciprocal-Intercross Progeny (F_2_) The parental strains were from Santa Rosa Island, Florida, United States (white circle) and Ocala National Forest, Florida, United States (brown circle). Based on quantitative measurements, parental subspecies differ significantly in seven pigmentation traits (Student's t-test, *p* < 0.001). For some traits, F_1_ hybrids are intermediate between the parental phenotypes (e.g., earbase pigmentation), while for others they are more similar to either the beach parents (e.g., lack of a tail stripe) or to mainland parents (e.g., extent of dorsal pigmentation), suggesting variation in the degree of additivity and dominance of alleles contributing to the light phenotype. The F_2_ progeny show continuous phenotypic variation in pigmentation patterns, consistent with a multigenic basis of patterning variation. However, parental phenotypes are recovered in the F_2_ progeny, suggesting that differences in pigmentation are controlled by a small number of genes.

We focused on the two subspecies of P. polionotus showing the greatest difference in color pattern*: P. p. subgriseus* and *P. p. leucocephalus.* The mainland subspecies *(P. p. subgriseus)* occupies oldfield habitats in the southeastern United States and has a coat that is dark brown on top and light gray on the belly, as well as a striped tail. In contrast, the light-colored Santa Rosa Island beach mouse *(P. p. leucocephalus),* like other “beach mice” that have colonized Florida's barrier islands and sandy coastal dunes, lacks visible pigmentation on its face, flank, and tail (Figure 1).

## Results/Discussion

**Figure 2:**
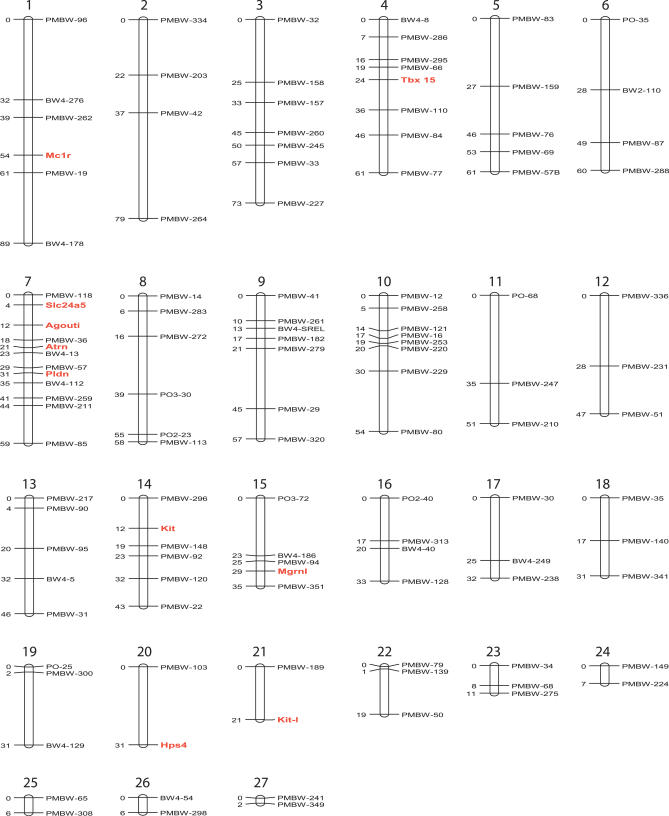
A Genome-Wide Linkage Map of P. polionotus Comprising 27 LGs Ordered by Size Microsatellite loci are indicated in black and candidate pigmentation genes in red. The cumulative genetic distance between markers is given in centimorgans (cM). All candidate genes were on separate LGs with the exception of *Pldn, Atrn, Slc24a5,* and *Agouti,* which clustered on LG 7. A total of three microsatellite loci and one pigmentation gene *Tyr* failed to show linkage to any other markers.

To analyze the genetic basis of color-pattern difference, we made reciprocal genetic crosses between three mainland and three beach mice, yielding 28 F_1_ hybrids that were then intercrossed to produce 465 F_2_ progeny. A genome-wide linkage map was generated using both anonymous microsatellite markers and single nucleotide polymorphisms (SNPs) in candidate pigmentation genes (Figure 2). This represents the first genome-wide linkage map for *Peromyscus,* with the exception of an allozyme-based map with few markers [7]. We scored all F_2_ progeny for 113 microsatellite markers fixed within but differing between the two subspecies (Table S1). We also scored F_2_ progeny for SNPs in 11 pigmentation genes chosen because of their chromosomal location and their known mutational effects on pigmentation in *Mus* (Tables S2–S4). In sum, we analyzed the linkage of 124 informative markers scored in all 465 F_2_ progeny (57,660 genotypes) using JoinMap software [8]. The markers were ordered in 27 linkage groups (LG) based on a log likelihood of odds (LOD) ratio of linkage threshold of 4.2 (permutation test, *p* = 0.05). The combined LGs span 1,103 cM (by comparison, the *Mus* genome comprises ∼1,300 cM [9]), with a mean interval length between markers of 8.9 cM. Because P. polionotus has 24 chromosomes [10], we expect that the markers will collapse into 24 LGs when additional regions are screened.

**Figure 3:**
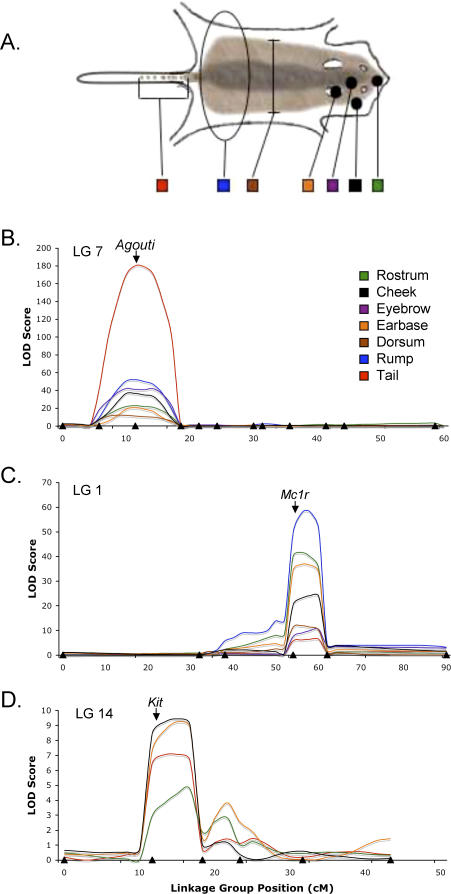
Genetic Architecture of Pigmentation in a Cross between Beach and Mainland Subspecies (A) Each of seven pigmentation traits for which we found significant QTLs is highlighted in a different color on a cartoon of a mouse pelt. MQM analyses showed that two LGs harbored major effect QTLs and one LG carried a minor effect QTL. For each LG, LOD scores are shown as a function of genetic distance in centimorgans (cM). Black triangles on x-axes show the position of marker loci. Each line indicates the LOD score at 5-cM intervals along the LG and are coded by colors corresponding to each of the seven traits. (B) One region of major affect maps to LG 7 and the *Agouti* locus maps to the peak in LOD score. (C) A second major-effect region is located on LG 1, and the *Mc1r* locus maps to the peak in LOD score. Both major-effect loci are statistically associated with all seven pigmentation traits studied. (D) A QTL of minor effect is located in LG 14, and the *Kit* locus maps near the peak. This minor effect locus is associated with four of the seven pigmentation traits.

To identify which genomic regions were statistically associated with the pigmentation differences, we determined the phenotypes of F_2_ progeny in seven regions of the body. These regions show the most divergence in pigmentation between the subspecies and together accurately encapsulate the difference in color and pattern. We measured total pelage reflectance (brightness) and scored pigment pattern on individual hairs for four facial traits (rostrum, cheek, eyebrow, and earbase) and also calculated the extent of dorsal, rump, and tail pigmentation as three additional traits (Figure 3A). The phenotypic data show no evidence for sexual dimorphism or maternal effects. The phenotypic correlation (*r*) between traits ranged from 0.29 to 0.82 (the highest value between earbase and cheek), suggesting that while some genes cause similar pigmentation differences among different body parts, other genes have more localized effects (Figure S1A). The distribution of phenotypic scores among F_2_ individuals was not consistent with simple Mendelian inheritance for any of the traits with the exception of tail stripe, which shows a bimodal distribution of scores (Figure S1B). We analyzed these phenotypic values, along with the molecular marker data, using MapQTL 5 [11].

Only three LGs harbored quantitative trait loci (QTL) that influence pigmentation differences between the subspecies (Figure 3B–3D). Because pigmentation has served as a model pathway for studies of gene action and interaction in a variety of biological processes, there are over 100 well-characterized genes known to affect pigmentation in laboratory mice [12,13]. Each of the three QTL regions contains only a single pigmentation gene from the homologous regions (bounded by homologous microsatellite markers) of the closely related model organisms Mus musculus and *Rattus norvegicus:* these genes are the *Agouti signaling protein* (*Agouti;* LG 7), the *melanocortin-1 receptor* (*Mc1r;* LG 1), and the c*-kit receptor* (*Kit;* LG 14). When mapped in *Peromyscus,* markers in these three candidate genes showed the highest LOD values for all seven pigmentation traits compared to other markers in the same LG (Table 1). Application of Multiple QTL model (MQM) mapping methods shows that none of the other eight candidate genes or 113 microsatellites is significantly associated with pigmentation variation. Our results suggest that nearly all of this difference is likely due to the three pigmentation genes *Agouti, Mc1r,* and *Kit,* although it is formally possible that other closely linked loci affect the color difference between subspecies. Below, we refer to these three QTLs using the names of the candidate genes.

**Table 1:**
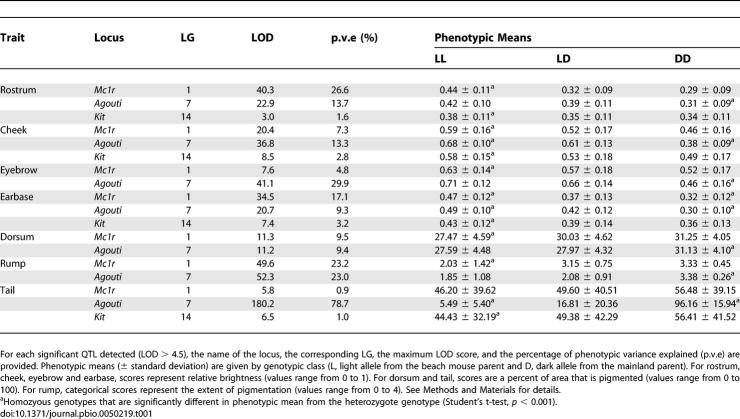
Location and Magnitude of QTLs Affecting Pigmentation Pattern

Each of the two regions of largest effect, *Agouti* and *Mc1r,* influence all seven pigmentation traits (LOD > 5.8). *Agouti* explains the greatest amount of pigment variation for three traits (cheek, eyebrow, and tail), while *Mc1r* explains the greatest amount of variation for two traits (rostrum and earbase). Both regions contribute equally to the extent of dorsal and rump pigmentation. The relative phenotypic effect of these two regions varies among traits (Table 1). For example, *Agouti* explains 78% of the variation in tail striping, but only 9% of the variation in dorsal pigmentation, while *Mc1r* explains 27% of the variation in rostrum pigmentation but only 1% of the variation in tail striping. Depending on the trait, the combination of these two loci explains between 19% and 80% of the variation for each of the pigmentation traits. The candidate gene *Kit* mapped to the only QTL of small effect, which explained less than 3.2% of the phenotypic variation among traits. This region is associated with only four traits (rostrum, cheek, earbase, and tail; LOD > 3.0), thus showing more spatial specificity than the two regions of major effect. The remaining phenotypic variance is likely attributable to other loci of small effect that are undetectable in a cross of this size and to environmental and/or epigenetic variation. Thus, a small number of chromosomal regions*—*and perhaps only a few genes*—*are responsible for most of the difference in color pattern between subspecies.

One of the classical ways to determine the effects of genetic variation on pigmentation is to analyze the allelic composition of extreme classes in an F_2_ or backcross (e.g., 14). An analysis of the most extreme phenotypes among our F_2_ progeny shows a striking association between phenotype and the allelic variation (“light” allele derived from the beach parents [L] and “dark” allele derived from the mainland parents [D]) at *Agouti* and *Mc1r*. Of the 50 F_2_ progeny with the lightest dorsal pigmentation, 42 had at least one light *Mc1r* allele (LL or LD *Mc1r* genotypes; χ^2^ test, *p* < 0.0001). Similarly, of the 113 F_2_ progeny lacking a tail stripe, 112 had at least one light *Agouti* allele (LL or LD *Agouti* genotypes; χ^ 2^ test, *p* < 0.0001).

The direction and magnitude of QTL effects were gauged by comparing phenotypic means among the F_2_ offspring. For the two major QTLs, the derived *Agouti* and *Mc1r* alleles increase the average coat reflectance (i.e., produce lighter color) and reduce the extent of dorsal, rump, and tail pigmentation (Table 1), changes consistent with the idea that these alleles were fixed by natural selection in beach mice. In addition, population-specific alleles of *Mc1r* and *Agouti* show differences in dominance for all traits. For example, 30 F_2_ progeny lack pigmentation on the rump. Among these mice, the distribution of *Mc1r* alleles (DD = 0, DL = 2, and LL = 28) suggests that the light *Mc1r* allele is largely recessive. By comparison, the distribution of *Agouti* alleles in the same 30 F_2_ progeny (DD = 0, DL = 15, and LL = 15) suggests that the light *Agouti* allele is dominant to the dark allele. These patterns are consistent with the dominance hierarchy of these genes seen in laboratory mice, in which either recessive loss-of-function mutations in *Mc1r* or dominant gain-of-function mutations in *Agouti* yield lighter pigmentation [15,16].

Mc1r is an integral membrane protein of melanocytes, which are pigment-producing cells. Agouti, the ligand of Mc1r, is an inverse agonist that, when bound, reduces Mc1r activity (via lowered cAMP signaling) resulting in lighter pigmentation. Thus, it is the biochemical interaction between these two proteins that controls the switch between dark eumelanin and light phaeomelanin production in melanocytes [17]. Previous work showed that laboratory populations of beach and mainland mice differ by a fixed, single amino acid mutation that reduces *Mc1r*'s signaling potential [18], but additional changes in *Mc1r* expression levels have not been ruled out as a contributor to the difference in pigmentation (see below).

To identify whether a coding change in the *Agouti* gene itself might also contribute to the pigmentation differences between our subspecies, we sequenced *Agouti'*s three translated exons (encoding a 139 amino acid protein) in the six original parents of our cross (Figure S2). The beach and mainland sequences did not differ by any fixed nonsynonymous mutations, demonstrating that amino acid changes in *Agouti* are not responsible for the color differences. In addition, sequencing of *Agouti* cDNA products showed that both mainland and beach mice produced an intact and spliced *Agouti* transcript similar to that observed in *Mus*. Because most of the *Agouti* mutations that produce light coloration in laboratory mice involve gain-of-function *cis*-regulatory mutations [19], we also tested the prediction that an increase in *Agouti* expression contributes to the light coloration of beach mice.

**Figure 4:**
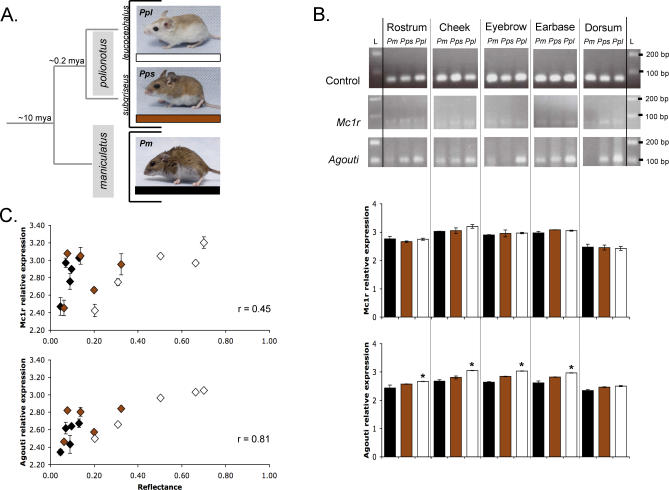
Gene Expression Assays for *Mc1r* and *Agouti* in Five Pigmentation Regions in Three *Peromyscus* Taxa (A) Phylogenetic relationship and approximate divergence times are shown for the P. polionotus subspecies and the sister species P. maniculatus. Photos show typical pigmentation pattern for each taxon. The phylogeny and pictures together highlight the similarity in pigmentation patterns between the mainland P. polionotus and *P. maniculatus,* the close evolutionary relationship between P. polionotus subspecies, and the derived nature of the beach mouse phenotype. (B) Qualitative (RT-PCR) and qPCR expression levels of *Mc1r* and *Agouti* mRNA relative to *beta-Actin* (control gene) for five distinct tissue samples in P. polionotus subspecies (*P. p. leucocephalus* [*Ppl*; white] and *P. p. subgriseus* [*Pps*; brown]) and P. maniculatus (*Pm*; black) are shown. A 100-bp ladder (L) flanks both sides of the RT-PCR gels. For the qPCR assays, since low Ct values indicate high expression level, we transformed the raw expression data to be more intuitive: relative expression values represent the averaged Ct values for each species subtracted from the sum of expression values across all species. Significant differences in relative expression levels between P. polionotus subspecies are indicated by asterisks (Student's t-test, *p* < 0.05). Bars indicate the standard error for each assay. (C) Association between level of *Mc1r* and *Agouti* relative expression and pigmentation (measured by reflectance) is shown among the three taxa*.* Correlation (*r*) values are shown.

To examine whether differences in expression level of *Mc1r* or *Agouti* influence color patterning, we conducted gene expression assays (reverse transcriptase PCR [RT-PCR] and quantitative-PCR [q-PCR]) on adult skin taken from five of the seven assayed pigmentation areas (Figure 4). Specifically, we performed parallel expression analyses for *Mc1r, Agouti,* and *beta-Actin* (a ubiquitously expressed control gene) mRNAs in the two *polionotus* subspecies. We also included mRNA from their fully pigmented sister species *P. maniculatus,* to determine which subspecific expression pattern is derived and which is ancestral. As a control, we compared patterns of *Mc1r* and *Agouti* expression in skin taken from the dorsum, a region that shows similar levels of pigmentation in beach and mainland subspecies and thus should show little difference in *Mc1r* and *Agouti* expression. Dorsal skin showed no significant difference in *Mc1r* expression among the three taxa (analysis of variance, *p* = 0.96). *Agouti* expression on the dorsum did not differ significantly between beach and mainland subspecies (*p* = 0.13), but *Agouti* was expressed at a lower level in P. maniculatus than in either *polionotus* subspecies (*p* < 0.01), consistent with P. maniculatus's darker dorsal pigmentation.

We also compared levels of *Mc1r* expression between the three taxa for four body regions (rostrum, cheek, eyebrow, and earbase) that show large differences in pigmentation between beach and mainland mice. There was no difference in *Mc1r* expression level among taxa or among body regions (analysis of variance, *p* > 0.05) and no correlation between *Mc1r* expression level and reflectance among taxa across body regions when all taxa were included (Figure 4; *r* = 0.45, *R* ^2^ = 0.20, and *p* = 0.10). Thus, taken together with earlier functional analyses [18], these data suggest that a single amino acid mutation in the coding region of *Mc1r*—and not mutations in neighboring *cis*-regulatory regions*—*produces light pigmentation in beach mice. Finally, in the same four body regions, *Agouti* expression was always significantly higher in tissues from beach mice than in tissues from mainland mice (Student's t-test, *p* < 0.05, two-tailed test). Comparing *Agouti* expression in P. polionotus to its sister species *P. maniculatus,* we find that the increased expression in beach mice is a derived trait because both *P. p. subgriseus* and P. maniculatus have similarly low levels of *Agouti* expression. In addition, *Agouti* expression is significantly correlated with pelage reflectance when all three taxa are compared (Figure 4C; *R* ^2^ = 0.65, and *p* < 0.001). *Agouti* also explains spatial variation in light coloration within a subspecies; there is a significant positive correlation between pelage reflectance and *Agouti* expression across body regions in beach mice (*R* ^2^ = 0.91, and *p* < 0.05). Together, these results suggest that increased expression of *Agouti*, caused by either mutation(s) in its *cis*-regulatory region or in closely linked *trans-*factors, also contributes to the light phenotype of beach mice.

**Figure 5:**
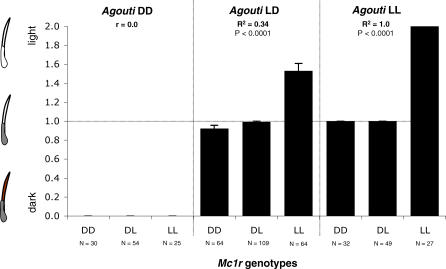
Interaction between *Agouti* and *Mc1r* Genes for One Trait, Cheek Pigmentation Black bars represent the mean phenotypic score for each *Mc1r* genotype when F_2_ progeny were clustered by *Agouti* genotype; alleles are indicated by dark (D) and light (L). Phenotypic values were taken for each of 465 F_2_ progeny, scored as 2 (no visible pigment), 1 (partially pigmented), and 0 (fully pigmented) as indicated by the cartoons of individual hairs. Sample size and standard error bars are provided. *R* ^2^ and *p*-values obtained by χ^2^ test are shown for each *Agouti* genotype, indicating the amount of phenotypic variation explained by *Mc1r* genotype in the F_2_ progeny.

Because the Mc1r and Agouti proteins interact physically, we tested for epistasis by performing gene interaction analyses (MapManager QTXb [20]). We found evidence of epistasis in several pigmentation traits (e.g., eyebrow: LOD score = 11.28; χ^2^ test, *p* = 0.001 and rostrum: LOD score = 10.32; χ^2^ test, *p* = 0.001). We also examined the effect of *Mc1r* genotypes on different *Agouti* backgrounds (and vice versa) using a categorical measurement of pigmentation. We detected epistasis for all seven of the traits but most strikingly for cheek pigmentation (Figure 5): F_2_ mice with the *Agouti* DD genotype always have fully pigmented hairs regardless of *Mc1r* genotype. In fact, for all seven traits there is no difference in phenotype between individuals who have either the DD or DL *Mc1r* genotypes on an *Agouti* DD genetic background (Student's t-test, *p* > 0.05), and for only a few traits (e.g., tail) is the *Mc1r* LL genotype visible on the *Agouti* DD background. In contrast, in F_2_ mice having the *Agouti* LL genotype, the *Mc1r* genotype explains all of the variation in cheek pigmentation (*r* = 1.0 and *p* < 0.0001); and double mutants (*Agouti* LL and *Mc1r* LL) always lack visible pigment in their cheek hairs (Figure 5). Clearly, *Mc1r* genotype has a significant effect on pigmentation only on the light *Agouti* (LL and LD) background, a pattern mirrored in all seven traits. Thus, the effect of each of the two genes on phenotype clearly depends on the genotype at the other locus.

It is striking that the interaction between *Agouti* and *Mc1r* in these mice is just the reverse of what we would predict from the classical genetics of the laboratory mice. In the pigmentation pathway, *Mc1r* is downstream of *Agouti* action, and in the laboratory variation in *Mc1r* has been shown to mask the action of *Agouti* alleles [21,22]. In contrast, we show here that *Agouti* can mask *Mc1r,* even though the dominance hierarchy of alleles remains identical to that seen in laboratory mice. We can explain this pattern of “reverse epistasis” mechanistically. The single mutation in *Mc1r* significantly decreases agonist (αMSH) binding, and hence cAMP signaling, but does not eliminate the receptor functionality of its protein product [18]. However, it is only with increased expression of *Mc1r*'s antagonist *Agouti* that the phenotypic effects of this weakened *Mc1-receptor* are revealed, a result consistent with *Agouti*'s ability to decrease cAMP production independent of αMSH. Thus, it is clear that epistasis is a property of particular alleles rather than of loci themselves, and thus epistatic interactions observed in the laboratory may differ from those seen in natural populations [see 23].

In sum, our genome-wide linkage map for *Peromyscus* allowed us to identify three genetic regions, two of which have major phenotypic effects on the adaptive difference in color pattern between subspecies of P. polionotus. Moreover, these regions contain the well-studied *Agouti* and *Mc1r* pigmentation genes [13]. While mutations in *Mc1r* are correlated with pigmentation in a number of wild vertebrates [see 13], our results are, to our knowledge, the first example of variation at the well-studied *Agouti* locus being associated with adaptive variation of animal coloration in nature.

Our results also have several implications for understanding the genetic basis of adaptation. First, this subspecific difference in color pattern is produced by a few interacting genes of large effect, supporting the idea that adaptations can involve relatively few genes rather than, as is often believed, many genes of small effect [24]. When an animal suddenly invades a novel habitat, their ancestral phenotype is often very different from the new optimal phenotype (as was almost certainly true for beach mice). Indeed, population genetic theory predicts that mutations of large effect will often be involved in adaptation in these circumstances [25], a prediction consistent with several studies on the genetic basis of mimicry and crypsis [26–28]. Second, both structural mutations (a single amino acid change reducing *Mc1r* signaling potential) and regulatory mutations (a derived increase in *Agouti* expression) contribute to adaptive change, and this change involves both recessive (*Mc1r* L) and dominant (*Agouti* L) alleles. These results support the idea that adaptation is not necessarily driven largely by *cis*-regulatory changes [29,30] or by (semi) dominant alleles [31,32]. Third, we show that the nature of epistasis between *Mc1r* and *Agouti* in wild populations does not mirror that seen in the laboratory, suggesting that one should be cautious not only about extrapolating the genetics of laboratory strains to evolution in nature, but also about inferring the directionality of biochemical pathways from patterns of gene interactions. Finally, most genetic studies of morphological change have concentrated on the loss of phenotypic traits through loss-of-function mutations (e.g., reduced armor in stickleback fish [33,34], absence of wing spots in *Drosophila* [35], and lack of pigment in cavefish [36]). This study provides a novel example of how adaptation can result from mutations involving a gain of function.

## Materials and Methods

### Genetic crosses.

Parental stocks were maintained at the *Peromyscus* Genetic Stock Center (University of South Carolina, United States). Maintenance of stocks and the crossing design have been described previously [18].

### Phenotyping.

A total of seven pigmentation traits (rostrum, cheek, eyebrow, earbase, and the extent of pigmentation on the dorsum, rump, and tail) were scored in all 465 F_2_ individuals. A spectrophotometer (Ocean Optics, http://www.oceanoptics.com) was used to capture reflectance spectra from the four facial traits (rostrum, cheek, eyebrow, and earbase). A reflectance probe was held at a 45 ° angle to the surface, and the program OOIbase32 (Ocean Optics) was used to capture reflectance measurements from 300–700 nm. Brightness was calculated by summing the area under the reflectance curve and converting to a normalized reflectance [37]. The extent of dorsal and tail pigmentation was measured as the proportion of the area that was pigmented. Rump pattern was scored using five categories from minimally (0) to fully (4) pigmented (following [1–3]). In addition, categorical pigmentation values (0–2) were scored for all seven pigmentation traits in the F_2_ progeny (following [18]). All statistical correlation analyses for the color traits were performed using JMP version 5.1.2 statistical software package (SAS Institute, http://www.sas.com).

### Genotyping assays.

All F_2_ individuals were genotyped for a total of 113 anonymous microsatellite markers and 11 SNPs in pigmentation genes.

Microsatellites were cloned from enriched partial genomic libraries developed for *P. maniculatus bairdii* and *P. polionotus subgriseus* [38]. Cloned sequences were edited in Sequencher 3.1.1 (Genecodes, http://www.genecodes.com), and microsatellite motifs were identified by eye. PCR primers designed to amplify the repeat motifs were used to genotype the six parental mice (three beach and three mainland parents). Out of 400 microsatellite loci tested, 113 showed diagnostic differences between individuals from the two subspecies, and these were scored in the 465 F_2_ progeny. All microsatellite loci were inherited in a codominant manner and were anonymous (with the exception of one microsatellite identified in a pigmentation gene, t*-box protein 15* [*Tbx 15*]). Microsatellite markers used to construct the linkage map are listed in the Table S1.

All PCRs were performed in a 15 μl volume using Eppendorf Mastercycler Gradient thermal cyclers (http://www.eppendorf.com). Each reaction included 30 ng of template DNA, 10× Taq Buffer with 1.5 mM MgCl2 (Eppendorf), 0.3 μL of 10 mM dNTPS, 0.6 μM each of a fluorescently labeled forward primer, unlabeled reverse primer, and 0.15 units Taq DNA polymerase (Eppendorf). The majority of microsatellite primers were synthesized with a known CAG (5′-CAGTCGGGCGTCATCA-3′) or M13R sequence (5′-GGAAACAGCTATGACCAT-3′) attached to the 5′ end. The PCR master mixes used in this system included 0.06 μM of the sequence-tagged primer, 0.6 μM of the untagged primer, and 0.54 μM of the fluorescently labeled probe.

The cycling conditions for all primer pairs followed a touchdown protocol (successively lower annealing temperatures). PCR parameters were: 94 °C for 90 s, followed by 21 cycles of denaturation at 94 °C for 30 s, 55 °C annealing for 30 s, and 72 °C for 1 min. The initial annealing temperature decreased by 0.5 °C for each of 20 cycles. An additional 15 cycles were performed as follows: 94 °C for 30 s, followed by 30 s at the last temperature, and 72 °C for 1 min. The final extension occurred at 72 °C for 5 min.

Amplification products were scored on an ABI 3100 (http://www.appliedbiosystems.com) in a 96-well format and genotyping was multiplexed by labeling loci with different 5′ fluorescent dyes: 6-FAM (blue), VIC (green), and NEB (yellow). Rox Genescan 400HD (Applied Biosystems) was used as internal size standard, and PCR products were analyzed with Genemapper version 3.5 software (Applied Biosystems).

In addition to microsatellite markers, 11 candidate pigmentation genes were screened for SNPs that were diagnostic between the two P. polionotus subspecies (Table S2). Candidate genes were chosen based on their known phenotypic effects, both on pigmentation and pleiotropic effects on other traits, in laboratory mice. For each candidate gene, PCR primers were designed in conserved exonic regions based on alignments of mouse, rat, and human sequences. Amplification primers were designed to span introns to maximize variation between subspecies. Following PCR optimization, introns were amplified in all six parents to identify diagnostic polymorphisms. Sequences were edited using Sequencher, and diagnostic markers were identified by eye. PCR primers and amplification conditions are listed in Table S3.

Genotyping of three candidate loci, *Kit, Kitl,* and *Hps4,* was performed using a restriction enzyme digest assay. One microgram of *Kit* amplification product was digested at 60 °C for 60 min with 1 unit of BsiEI, 1× NEBuffer, and 100× BSA (10 mg/ml) in a total reaction volume of 50 μL. *Kitl* and *Hps4* amplicons were digested in a total reaction volume of 15 μl at 37 °C for 4 h using Hpy188 III and PspOM I enzymes, respectively. Digestion products were visualized on a 1.5% agarose gel stained with ethidium bromide.

A polymorphic microsatellite was identified in the first intron of *Tbx 15*. Genotyping of *Tbx 15* was scored on an ABI 3100 in a 96-well format. Genotyping of seven candidate pigmentation genes *(Mc1r, Agouti, Tyr, Atrn, Slc24a5, Pldn,* and *Mgrn1)* was performed on an ABI 7000 using a TaqMan assay. A total of 60 ng of genomic DNA was used in each reaction, and cycling parameters were as follows: 40 cycles of 50 °C for 2 min, 95 °C for 10 min, and 92 °C for 15 s followed by an allelic discrimination step of 60 °C for 2 min. The TaqMan primer sequences are listed in Table S4.

### Linkage map construction.

A genetic linkage map was generated using JoinMap version 3.0 [8] on a locus file containing genotypes of a total of 124 molecular markers in 465 F_2_ progeny, with the population type set for segregation of two alleles per locus (F_2_ population). JoinMap was used with an LOD score threshold of 6.0 to assign 120 of 124 loci to 27 LGs. For each LG, a map was created considering: Kosambi mapping function, default LOD (1.0) and recombination (0.4) thresholds, jump threshold of 5.0, and not fixed order. A ripple analysis was performed after all markers on the LG were added to the map. This analysis attempts to improve the order of the loci in a chromosome by testing alternative orders created by local permutations of the locus order.

### QTL mapping.

All quantitative measures of pigmentation traits were analyzed with MapQTL 5 [11] using the interval mapping (IM) method, which fits a single QTL model (additive versus dominant model). Using likelihood ratio tests in MapManager QTXb [20], we verified that the additive versus dominance model was the best model of allelic effects. Similar mapping results were observed for the quantitative and categorical datasets**.** The MapQTL 5 parameters used were: mapping step size of 2.0 cM, maximum of 200 interactions, functional tolerance value of 1.0e^−8^, and a minimum of five flanking markers to resolve incomplete genotypes. MQM mapping was performed in LGs where several QTLs were detected. Cofactors for MQM analyses were automatically selected with a *p*-value of 0.02. Results from MQM analyses improved initial IM outputs by identifying from multiple to a single QTL per LG. Significance thresholds for determining linkage were chosen using conservative criteria for genome-wide linkage mapping in noninbred individuals: significant linkage of LOD ≥ 4.5 [39]. Significance of LOD values for each trait was confirmed by permutation tests in MapQTL 5, with a genome-wide significance level of α = 0.05 and 1,000 iterations. Calculation of the percentage of phenotypic variance explained (p.v.e) by a QTL was performed in MapQTL 5 on the basis of the population variance found within the progeny of the cross.

Gene interaction analyses to identify epistasis between QTLs were performed using MapManager QTXb. Probability of association was set at *p* = 0.0001, and the LOD thresholds for each quantitative trait were estimated by permutation tests.

### RT-PCR and qPCR.

Total RNA was isolated for four facial regions and the dorsum from shaved adult skin tissue of *P. polionotus leucocephalus*, *P. p. subgriseus,* and P. maniculatus using TRIzol reagent (Invitrogen, http://www.invitrogen.com) following the manufacturer's protocol. Total RNA was treated using DNase I (New England BioLabs, http://www.neb.com). Subsequent reverse transcriptase reactions were performed using the Titan One tube RT-PCR kit (Roche, http://www.roche.com) with specific *Peromyscus* primers of *Agouti* (forward 5′-TCTCTGGTGGGTGGGACTTC-3′ and reverse 5′-TGATTTTAGCCTCCATTAGGTTTCC-3′; exons 2–4), *Mc1r* (forward 5′-TGGACATACAGAATTGCCATGAG-3′ and reverse 5′-CAACCACACAGCCGTCCTAA-3′; exon 1), and *beta-Actin* (forward 5′-TCCTGACTGAGCGTGGCTATAG-3′ and reverse 5′-TCTCTTTGATGTCACGCACGAT-3′; exon 4) genes. Although *Agouti* has two differentially expressed transcripts, these primers were designed to measure expression of both isoforms simultaneously. For all experiments, both no-RT and no-Template controls were included.

PCR products were visualized on a 1.5% agarose gel. RT-PCR were also performed using the SuperScript III Reverse Transcriptase (Invitrogen), RNaseOUT (Invitrogen), and the oligo(dT)_20_. qPCR amplifications were conducted in 20 μl reactions containing approximately 100 ng of total cDNA, 10 μl 2× TaqMan Universal PCR Master Mix, and 1 μl 20× TaqMan gene expression assay of *Agouti*, *Mc1r,* and *beta-Actin*. The amplification protocol used was as follow: initial 10 min denaturation at 95 °C followed by 50 cycles of 95 °C for 15 s and 60 °C for 1 min. Amplification signals were detected continuously with an ABI 7000 sequence detection system. All expression assays were done in either duplicate or triplicate.

Analysis of the qPCR data was conducted by calculating the average Ct value across replicate experiments for each target gene *(Mc1r* and *Agouti)* and normalizing by the average Ct value of the reference gene *(beta-Actin)* for a specific tissue. Significance of the qPCR data was determined by one-way ANOVA and Student's t-tests using the JMP statistical package.

## Supporting Information

Figure S1 Pigmentation Traits Scored in F_2_ Progeny (A) Pair-wise correlations based on orthogonal regression analyses among all seven traits are shown. Comparisons within facial and body regions are boxed.
(B) The distribution of trait values among F_2_ offspring is presented. The mean phenotypic values for *P. polionotus subgriseus* (PO), *P. p. leucocephalus* (LS), and F_1_ individuals are indicated. For all traits, the mean phenotypic value of the parental subspecies (LS and PO) differs significantly (*p* < 0.001).
(848 KB PPT)

Figure S2 Genomic Structure of the *Agouti* Gene Including the Known *Cis*-Regulatory and Coding Regions Based on the Comparison of *Mus* and *Peromyscus* BAC Sequences DNA sequence alignment of the complete translated region of *Agouti* (exon 2 = 170 bp, exon 3 = 89 bp, and exon 4 = 390 bp) from M. musculus (*Mm*), P. maniculatus (*Pm*)*, P. p. subgriseus* (*Pps*), and *P. p. leucocephalus* (*Ppl*). Gray squares are known *cis*-regulatory elements of the gene; black squares are coding regions of the gene. The exon junctions are indicated by black triangles. The single polymorphic synonymous mutation between *Pps* and *Ppl* is indicated by a gray triangle.
(27 KB PPT)

Table S1 Microsatellite Markers Used to Generate P. polionotus Linkage Map (92 KB DOC)

Table S2 Candidat Pigmentation Genes Gene name, symbol, phenotypic effect in *Mus,* functional role, *Mus* chromosome, *Peromyscus* LG, marker type (e.g., microsatellite, RFLP or SNP), and the location of the marker in *Peromyscus* (e.g., intron and exon), are provided.
(74 KB DOC)

Table S3 PCR Primers and Conditions Used to Amplify Candidate Pigmentation Genes Sequences of the forward and reverse primers used to amplify part of each gene are shown as well as PCR conditions (e.g., the annealing temperature and extension time).
(70 KB DOC)

Table S4 SNP Genotyping at Candidate Pigmentation Genes Sequences of the forward and reverse primers and the fluorescently labeled probes are shown for each gene. SNPs were labeled using FAM or VIC dyes.
(54 KB DOC)

### Accession Numbers

The GenBank (http://www.ncbi.nlm.nih.gov/Genbank) accession numbers for the sequences discussed are: EU020066 (Peromyscus maniculatus), EU020067 (Peromyscus polionotus subgriseus), and EU020068 (Peromyscus polionotus leucocephalus).

## Abbreviations

*Agouti*: *Agouti signaling protein*
D: dark
*Kit*: *c-kit receptor*
L: light
LG: linkage group
LOD: likelihood of odds
*Mc1r*: *melanocortin-1 receptor*
MQM: Multiple QTL model
qPCR: quantitative PCR
QTL: quantitative trait locus
RT-PCR: reverse transcriptase PCR
SNP: single nucleotide polymorphism
