# Correction: Adaptive Variation in Beach Mice Produced by Two Interacting Pigmentation Genes

**DOI:** 10.1371/journal.pbio.0060036

**Published:** 2008-02-26

**Authors:** Cynthia C Steiner, Jesse N Weber, Hopi E Hoekstra

Correction for:

Steiner CC, Weber JN, Hoekstra HE (2007) Adaptive variation in beach mice produced by two interacting pigmentation genes. PLoS Biol 5(9): e219. doi:10.1371/journal.pbio.0050219

Figure S2 was erroneously labeled. The caption should read:

Figure S2. Genomic Structure of the Agouti Gene Including the Known *Cis*-Regulatory and Coding Regions (Based on Comparisons of Mammalian BAC Sequences: Mouse, Dog, Human)

In addition, there was an omission in the acknowledgments. The following sentence should be included:

T. Glenn, N. Schable, and C. Hagen produced microsatellite libraries and provided PmBW clone sequences that were funded by National Institutes of Health grant R01Gm069601 (to M. Dewey and T. Glenn).

