# Supplementary material for: Adaptive Variation in Beach Mice Produced by Two Interacting Pigmentation Genes
Source: PLoS Biol. 2007 Aug 14;5(9):e219. doi: 10.1371/journal.pbio.0050219 (PMC1945039; doi:10.1371/journal.pbio.0050219)
Supplement: Supplementary file 1 [file pbio.0050219.st001.doc]

**Table S1.** Microsatellite markers used to generate *Peromyscus polionotus* linkage map.

| Microsatellite | Microsatellite | Microsatellite | Microsatellite | Microsatellite | Microsatellite |
| --- | --- | --- | --- | --- | --- |
| *BW2-110** | PMBW-121 | PMBW-220 | PMBW-275 | PMBW-34 | PMBW-80 |
| *BW4-112** | PMBW-128 | PMBW-223 | PMBW-279 | PMBW-341 | PMBW-83 |
| *BW4-129** | PMBW-139 | PMBW-224 | PMBW-283 | PMBW-349 | PMBW-84 |
| *BW4-13** | PMBW-14 | PMBW-227 | PMBW-286 | PMBW-35 | PMBW-85 |
| *BW4-178** | PMBW-140 | PMBW-229 | PMBW-288 | PMBW-351 | PMBW-87 |
| *BW4-186** | PMBW-148 | PMBW-231 | PMBW-29 | PMBW-36 | PMBW-90 |
| *BW4-249** | PMBW-149 | PMBW-238 | PMBW-295 | PMBW-41 | PMBW-92 |
| *BW4-276** | PMBW-157 | PMBW-241 | PMBW-296 | PMBW-42 | PMBW-94 |
| *BW4-40** | PMBW-158 | PMBW-245 | PMBW-298 | PMBW-50 | PMBW-95 |
| *BW4-5** | PMBW-159 | PMBW-247 | PMBW-30 | PMBW-51 | PMBW-96 |
| *BW4-54** | PMBW-16 | PMBW-253 | PMBW-300 | PMBW-57 | *PO-17** |
| *BW4-8** | PMBW-182 | PMBW-256 | PMBW-308 | PMBW-57B | *PO2-23** |
| *BW4-SREL** | PMBW-189 | PMBW-258 | PMBW-31 | PMBW-65 | *PO2-40** |
| PMBW-103 | PMBW-19 | PMBW-259 | PMBW-313 | PMBW-66 | *PO-25** |
| PMBW-110 | PMBW-203 | PMBW-260 | PMBW-32 | PMBW-68 | *PO3-30** |
| PMBW-113 | PMBW-210 | PMBW-261 | PMBW-320 | PMBW-69 | PO-35 |
| PMBW-118 | PMBW-211 | PMBW-262 | PMBW-33 | PMBW-76 | *PO3-72** |
| PMBW-12 | PMBW-217 | PMBW-264 | PMBW-334 | PMBW-77 | PO-68 |
| PMBW-120 | PMBW-22 | PMBW-272 | PMBW-336 | PMBW-79 |  |
|  |  |  |  |  |  |

*Microsatellite markers described in [38]
