# Supplementary material for: Adaptive Variation in Beach Mice Produced by Two Interacting Pigmentation Genes
Source: PLoS Biol. 2007 Aug 14;5(9):e219. doi: 10.1371/journal.pbio.0050219 (PMC1945039; doi:10.1371/journal.pbio.0050219)
Supplement: Supplementary file 2 [file pbio.0050219.st002.doc]

**Table S2.** Candidate pigmentation genes. Gene name, symbol, phenotypic effect in *Mus*, functional role, *Mus* chromosome, *Peromyscus* linkage group, marker type (e.g., microsatellite, RFLP or SNP), and the location of the marker in *Peromyscus* (e.g., intron and exon), are provided.

| *Name* | Symbol | Mouse phenotype | Funct. role ^a^ | Mouse Chrom. | LG Pero. | Marker type | Location |
| --- | --- | --- | --- | --- | --- | --- | --- |
| *Pallidin* | *Pldn* | Pallid (pa) | C | 2 | 7 | SNP | Intron 2 |
| *Attractin* | *Atrn* | Mahogany (mg) | D | 2 | 7 | SNP | Intron 8 |
| *Solute carrier family 24, member 5* | *Slc24a5* | Golden (gol^b1^)* | B | 2 | 7 | SNP | Intron 6 |
| *Agouti signaling protein* | *Agouti* | Nonagouti (a) | D | 2 | 7 | SNP | Intron 1 |
| *Melanocortin-1 receptor* | *Mc1r* | Extension (e) | D | 8 | 1 | SNP | Exon 1 |
| c*-Kit receptor gene* | *Kit* | Dominant white-spotting (W) | A | 5 | 14 | RFLP | Exon 11 |
| t*-box protein 15* | *Tbx 15* | Droopy ear (de) | A | 3 | 4 | Micro. | Intron 1 |
| *E3 ubiquitin ligase* | *Mgrn1* | Mahoganoid (md) | D | 16 | 15 | SNP | Intron 14 |
| *Hermansky-Pudlak syndrome gene 4* | *Hps4* | Light ear (le) | C | 5 | 20 | RFLP | Exon 11 |
| *Ligand for c-Kit receptor gene* | *Kitl* | Steel (S1) | A | 10 | 21 | RFLP | Intron 7 |
| *Tyrosinase* | *Tyr* | Albino (c) | B | 7 | Unlinked | SNP | Exon 1 |

**^a^**Functional classes: A = melanocyte development, B = components of melanosomes, C = melanosome construction, D = melanin synthesis and switching. * = phenotype identified in zebrafish (*Dario rerio*)
