# Supplementary material for: Adaptive Variation in Beach Mice Produced by Two Interacting Pigmentation Genes
Source: PLoS Biol. 2007 Aug 14;5(9):e219. doi: 10.1371/journal.pbio.0050219 (PMC1945039; doi:10.1371/journal.pbio.0050219)
Supplement: Supplementary file 3 [file pbio.0050219.st003.doc]

**Table S3.** PCR primers and conditions used to amplify candidate pigmentation genes. Sequences of the forward and reverse primers used to amplify part of each gene are shown as well as PCR conditions (e.g., the annealing temperature and extension time).

| Candidate gene | Forward primer  5’ – 3’ | Reverse primer  5’ – 3’ | Annealing temperature | Annealing extension time |
| --- | --- | --- | --- | --- |
| *Mc1r* | TCTGGGTTCTCTCAACTCCA | AAAGCATAGATGAGGGGGTC | 56.0 °C | 60 sec |
| *Agouti* | TGGACTTCTAAAGGTCAGGAC  TCTCAACCAAAGTCCCCTCTTCC | CCAAAGGCACATCCTCCTACC  TGAACATGAAAGAAGTGGAACCATTCTG | 61.6 °C | 30 sec |
| *Tyr* | AATAGGACCTGCCAGTGCTC | GCTGGGCTGAGTAAGTTAGG | 57.5 °C | 30 sec |
| *Atrn* | ACTGCCATTAAGGTATGTCC | TGGTCCTGTGATCATAAACAC | 58.0 °C | 45 sec |
| *Hps4* | CCCCAGAAGAAGCAGTAGG | GCTGGATGTATAGGTTCATAGG | 54.0 °C | 40 sec |
| *Mgrn1* | GCCAGCACCTCGTGTGTC | AGCCGTGTCCTCGTCAAG | 53.2 °C | 60 sec |
| *Kitl* | TCTTCCCACCCCATCATC | CGAAGTAGCAGCCACATAAAG | 55.0 °C | 60 sec |
| *Tbx 15* | CCGTGCCTTGGGAAAATAGGAA | GCATATATTCATGCACACACATG | 50.0 °C | 60 sec |
| *Kit* | ACCTGTCCTGGCTTCCTTTGTCTTGTT | CTCTCTTTCTGTTAAATGGGCACTTGC | 58.0 °C | 60 sec |
| *Slc24a5* | GTGGGTGTCACTGTTGCTT | GACCACAGTGGGTACAATC | 54.8 °C | 45 sec |
| *Pldn* | TGAAGGACTGATAGAGG | AATCTCTTGTTCCAGTG | 52.4 °C | 45 sec |
