# Supplementary material for: Adaptive Variation in Beach Mice Produced by Two Interacting Pigmentation Genes
Source: PLoS Biol. 2007 Aug 14;5(9):e219. doi: 10.1371/journal.pbio.0050219 (PMC1945039; doi:10.1371/journal.pbio.0050219)
Supplement: Supplementary file 4 [file pbio.0050219.st004.doc]

**Table S4.** SNP genotyping at candidate pigmentation genes. Sequences of the forward and reverse primers, and the fluorescently labeled probes are shown for each gene. SNP were labeled using FAM or VIC dyes.

| Candidate gene | Forward primer  5’ – 3’ | Reverse primer  5’ – 3’ | Probe | Light Allele | Dark Allele |
| --- | --- | --- | --- | --- | --- |
| *Mc1r* | AGAATGTGCTGGTCGTGATAGC | TGAAGGAATACATGGGCGAGTG | CAGGTTGC[G/A]GTTTT | A - 6-FAM | G - VIC |
| *Agouti* | CCCTCTTCCCAAATGACTCTGA | GGTGGTGTGCTGGCTAGTTTT | CTTGTGTCACAC[G/T]GACAT | G - VIC | T - 6-FAM |
| *Tyr* | CCTATGGTTCACATGTGCTACTGT | TCACTTTCCTAACTACCATTGTGCAA | CTGTTGAGAATA[G/A]AGAAAA | A - VIC | G - 6-FAM |
| *Atrn* | CCAGAAAAGAACACATGGAGTATACTACA | GGCCATAGCCCCCTTGT | ACAAGAGCACC[A/C]TGAGTG | A - 6-FAM | C - VIC |
| *Mgrn1* | GGAGGCTTAATGGGTAGTCTAGGA | GAAGAGCAGAGGCAGACTTACG | TTCCCTGC[T/C]CCTCTTG | C - 6-FAM | T - VIC |
| *Slc24a5* | GGATGCCTAACTCTCAACTACTTGAA | GTATGGTGACCTGATTGATCCTTCA | CTTGAAGAAAAGA[A/G]AAAGTA | A - VIC | G - 6-FAM |
| *Pldn* | CAGGAGAGGAGTAGGAAGTTAGCTT | GGTCGGACCAGACAACCTTAA | TTCGTTAACA[T/G]TTGGATTC | T - VIC | G - 6-FAM |
